# Advances in Clinical Oncology Research on ^99m^Tc-3PRGD2 SPECT Imaging

**DOI:** 10.3389/fonc.2022.898764

**Published:** 2022-05-31

**Authors:** Liming Xiao, Jun Xin

**Affiliations:** Department of Radiology, Shengjing Hospital of China Medical University, Shenyang, China

**Keywords:** ^99m^Tc-3PRGD_2_, integrin αvβ3 receptor, oncology, single-photon emission computed tomography (SPECT), molecular imaging

## Abstract

The integrin alpha(α)v beta(β)3 receptor is ubiquitous in malignant tumors and has a certain level of specificity for tumors. Technetium-99m hydrazinonicotinamide-dimeric cyclic arginyl-glycyl-aspartic acid peptide with three polyethylene glycol spacers (^99m^Tc-3PRGD_2_) can bind specifically to the integrin αvβ3 receptor with high selectivity and strong affinity. Thus, it can specifically mark tumors and regions with angiogenesis for tumor detection and be used in single-photon emission computed tomography (SPECT) imaging. This modality has good application value for diagnosing and treating tumor lesions, such as those in the lung, breast, esophagus, head, and neck. This review provides an overview of the current clinical research progress of ^99m^Tc-3PRGD_2_ SPECT imaging for tumor lesions, including for the diagnosis and differential diagnosis of tumors in different body parts, evaluation of related metastases, and evaluation of efficacy. In addition, the future clinical application prospects and possibilities of ^99m^Tc-3PRGD_2_ SPECT imaging are further discussed.

## Introduction

Cancer is currently the leading cause of death worldwide, with a global estimated 19.3 million new cancer cases and almost 10.0 million cancer deaths recorded in 2020 (1). Early detection, diagnosis, and treatment are key measures for reducing mortality attributed to malignant tumors and prolonging survival time. The integrin alpha(α)v beta(β)3 receptor is frequently involved in the occurrence and development of malignant tumors (2, 3); it mediates cell–cell and cell–extracellular matrix adhesion (4, 5) and is related to tumor angiogenesis and metastasis (3, 6–8). The integrin αvβ3 receptor is highly expressed in activated endothelial cells and proliferating tumor cells; however, it is either not expressed or expressed at very low levels in normal endothelial cells, dormant vascular cells, and other normal cells (3, 9) and has a certain level of specificity. Therefore, the integrin αvβ3 receptor is a valuable target for diagnosing and treating malignant tumors.

Polypeptides containing the arginine-glycine-aspartate (Arg-Gly-Asp [RGD]) sequence can bind specifically to the integrin αvβ3 receptor with high selectivity and strong affinity (10). Hence, these polypeptides can specifically demarcate lesions and their angiogenesis for tumor detection and have promising prospects for tumor diagnosis and treatment. Radiolabeled RGD peptides and their analogs have been intensively studied for their application in the non-invasive imaging of integrin αvβ3 receptor expression (11–13).

The technetium-99m hydrazinonicotinamide-dimeric cyclic RGD peptide with three polyethylene glycol spacers (^99m^Tc-3PRGD_2_) is a ^99m^Tc-labeled molecular probe used in nuclear medicine for single-photon emission computed tomography (SPECT). Its core ligand, hydrazinonicotinamide-3PRGD_2_, is a new type of RGD dimer that can bind specifically to the integrin αvβ3 receptor with high selectivity and affinity. In addition, ^99m^Tc-3PRGD_2_ has rapid blood clearance and a high level of safety with no adverse reactions having been observed in animal models and humans to date (14, 15). ^99m^Tc-3PRGD_2_ SPECT imaging is widely used in clinical research because of its high diagnostic performance and excellent cost-effectiveness, which further highlight its potential for clinical applications. Herein, we review the advances in clinical research on ^99m^Tc-3PRGD_2_ SPECT imaging for tumor lesions over the past decade.

## Lungs

Globally, lung cancer is the leading cause of cancer-related death (1). Conventional radiography and computed tomography (CT) offer limited accuracy for lung cancer diagnosis and are poor evaluation methods for lung cancer-related metastasis. 2-deoxy-2-(18F)fluoro-D-glucose (FDG) positron emission tomography (PET) has become the standard of care in the initial management of patients with lung cancer, especially for those with non-small cell lung cancer (16). However, false-positive FDG PET/CT results in nodal staging have been shown in patients with coexistent inflammatory or infectious diseases (17, 18). In addition, a study (19) reported that ^68^Ga-NOTA-PRGD_2_ PET/CT has similar sensitivity and higher specificity than ^18^F-FDG PET/CT for the detection and differentiation of lung lesions. Moreover, it is superior to ^18^F-FDG PET/CT in that it can be used to judge metastatic lymph nodes with higher specificity. Nevertheless, PET is a relatively expensive technology, and PET scanners are not widely available (20). These limitations can interfere with clinical use. ^99m^Tc-3PRGD_2_ SPECT may be a more advantageous imaging modality.

### Diagnosis and Differential Diagnosis

Zhu et al. (21) examined the effectiveness of ^99m^Tc-3PRGD_2_ SPECT imaging for the assessment of lung cancer and found that with a low ^99m^Tc-3PRGD_2_ background in the lungs and mediastinum, most lung malignancies were prominent on 1-h images with tumor-to-background ratios significantly higher than those in benign lesions. Furthermore, most lymph-node and bone metastases could also be detected. Thus, their findings suggested that ^99m^Tc-3PRGD_2_ SPECT imaging is a sensitive tool for detecting lung cancer (sensitivity, 93–97%). Wang et al. (22) also reported that ^99m^Tc-3PRGD_2_ SPECT/CT has certain value for the diagnosis of benign and malignant lung lesions. In the diagnosis of solitary pulmonary nodules, Ma et al. (23) demonstrated the feasibility of using ^99m^Tc-3PRGD_2_ SPECT imaging for the non-invasive differential diagnosis of solitary pulmonary nodules. These results showed that the sensitivity of ^99m^Tc-3PRGD_2_ SPECT imaging for malignant lesions was higher than that of CT, with CT interpretation, SPECT visual analysis, and SPECT semiquantitative analysis showing sensitivity/specificity of 80/67%, 100/67%, and 100/67%, respectively. All solitary pulmonary nodules classified as indeterminate with CT were unequivocally diagnosed with ^99m^Tc-3PRGD_2_ SPECT imaging. Furthermore, immunohistochemical findings confirmed that integrin αvβ3 was expressed in nodules showing ^99m^Tc-3PRGD_2_ uptake. Zhang et al. (24) also showed that ^99m^Tc-3PRGD_2_ SPECT imaging could more accurately detect malignant solitary pulmonary nodules (the sensitivity/specificity of CT interpretation, SPECT visual analysis, and SPECT semiquantitative analysis were 82.4/71.4%, 100/71.4%, and 100/71.4%, respectively). Li et al. (25) showed that chest thin layer CT has an incremental value over ^99m^Tc-3PRGD_2_ SPECT/CT imaging for the differential diagnosis of benign and malignant pulmonary lesions. In a comparison between the diagnostic performance of ^99m^Tc-3PRGD_2_ SPECT imaging and ^18^F-FDG PET/CT imaging for lung tumors, Jin et al. (26) found no significant difference between the two methods (z=0.82; p=0.410). Moreover, their experiment further confirmed that the intratumoral accumulation level of ^99m^Tc-3PRGD_2_ was positively correlated with integrin αvβ3 expression (r=0.84; p=0.001) and microvessel density (r=0.63; p=0.011).

### Evaluation of Metastasis

Miao et al. (27) evaluated the diagnostic performance of ^99m^Tc-3PRGD_2_ SPECT imaging and conventional ^99m^Tc-MDP bone scanning for bone metastasis in patients with lung cancer. They reported that ^99m^Tc-MDP bone scanning provided better contrast for most lesions, whereas ^99m^Tc-3PRGD_2_ SPECT imaging appeared to be more effective at excluding pseudo-positive lesions and detecting osteolytic bone metastasis. A study by Shao et al. (28) further confirmed that the targeted binding of ^99m^Tc-3PRGD_2_ with integrin αvβ3 receptors on tumor cells and osteoclasts offers certain advantages for the early detection of osteolytic bone metastasis in lung cancer (the diagnostic sensitivity of ^99m^Tc-3PRGD_2_ SPECT imaging and ^99m^Tc-MDP imaging for osteolytic bone metastasis was 84.8% and 25.0%, respectively). Thus, ^99m^Tc-3PRGD_2_ SPECT imaging can serve as an effective supplement to conventional ^99m^Tc-MDP bone scanning. For the evaluation of lymph-node metastasis in non-small cell lung cancer, Jin et al. (26) demonstrated that ^99m^Tc-3PRGD_2_ SPECT imaging had a high diagnostic specificity (94.6%), which can compensate for deficiencies in the specificity of ^18^F-FDG PET/CT, and hence is of significant value for surgical decision-making in lung cancer.

### Evaluation of Efficacy and Prognosis

Ma et al. (29) examined the feasibility of using ^99m^Tc-3PRGD_2_ SPECT imaging to predict the efficacy of chemoradiotherapy plus bevacizumab in the treatment of advanced non-squamous non-small cell lung cancer. Their findings suggested that after two cycles of chemoradiotherapy plus bevacizumab administration, ^99m^Tc-3PRGD_2_ SPECT imaging could predict the patient’s treatment response and prognosis. A study by Zhang et al. (30) confirmed the feasibility of using ^99m^Tc-3PRGD_2_ SPECT imaging to evaluate the early response to treatment with epidermal growth factor receptor tyrosine kinase inhibitors among patients with advanced lung adenocarcinoma (sensitivity, 80.0%; specificity, 87.5%), as well as for predicting the prognosis and progression-free survival. Chen et al. (31) confirmed that ^99m^Tc-3PRGD_2_ SPECT scanning is a promising modality for predicting the tumor response in patients with advanced non-small cell lung cancer early in the course of bevacizumab therapy (sensitivity, 81.8%; specificity, 91.7%; negative predictive value, 84.6%). In addition, Yang et al. (32) explored the use of ^99m^Tc-3PRGD_2_ SPECT imaging for the evaluation of the clinical effect of recombinant endostatin (Endostar) for the inhibition of tumor angiogenesis. They concluded that since ^99m^Tc-3PRGD_2_ SPECT imaging compares the lesion T/N value (uptake ratio of the tumor to that of the normal contralateral lung tissue) before and after treatment, as well as the changes in the difference between the T/N values, ^99m^Tc-3PRGD_2_ SPECT has some clinical value in evaluating the efficacy of recombinant endostatin administration. Moreover, the T/N value was also significantly correlated with patient prognosis (r=0.879, p<0.001).

## Breast

Breast cancer is the most common malignancy among women worldwide and a major cause of cancer-related death among women aged 20–59 years (1). Early detection and diagnosis can substantially reduce breast cancer mortality. Conventional mammography is not ideal for identifying malignant tumors and has a high false-negative rate (33, 34). Magnetic resonance imaging and ultrasonography have limited value in differentiating benign abnormalities from malignancies (35). ^18^F-FDG PET/CT plays a very important role in the diagnosis and treatment of breast cancer (36, 37). Nevertheless, ^18^F-FDG PET/CT application is significantly restricted owing to high cost and low availability, as PET is a relatively expensive technology and neither PET scanners nor the cyclotrons required to produce isotopes for PET are widely available (20). Therefore, ^99m^Tc-3PRGD_2_ SPECT imaging could be an excellent alternative.

### Diagnosis and Differential Diagnosis

In a study exploring the diagnostic value of ^99m^Tc-3PRGD_2_ SPECT imaging for palpable and non-palpable breast lesions, Liu et al. (38) found that ^99m^Tc-3PRGD_2_ SPECT imaging had greater diagnostic value than mammography for the overall evaluation of breast lesions, showing sensitivity, specificity, accuracy, positive predictive value, and negative predictive value of 83/78%, 73/61%, 77/68%, 69/59%, and 85/80%, respectively. This modality may also enable response monitoring of breast cancer therapy through longitudinal imaging. Li et al. (39) conducted a comparative study on the value of ^99m^Tc-3PRGD_2_ SPECT/CT and conventional ultrasonography in distinguishing benign and malignant breast lesions and proved that ^99m^Tc-3PRGD_2_ SPECT/CT can distinguish benign and malignant breast lesions from an angiogenesis perspective with anatomical and functional information. Moreover, this modality can further identify benign and malignant lesions to reduce unnecessary punctures when ultrasonography is unavailable. Liu et al. (40) found that the T/NT ratio is related to the TNM staging of breast cancer, lymph-node metastasis, and HER-2. In addition, Ma et al. (41) compared the diagnostic performance of ^99m^Tc-3PRGD_2_ SPECT imaging and Tc-^99m^-MIBI SPECT imaging for malignant breast lesions and found that ^99m^Tc-3PRGD_2_ SPECT imaging did not offer significant advantages for the detection of primary breast cancer over Tc-^99m^-MIBI SPECT imaging (area under the receiver operating characteristic curve: 0.851 vs. 0.781; p>0.05). Furthermore, based on a comparison between the diagnostic performance of ^99m^Tc-3PRGD_2_ SPECT imaging and ^18^F-FDG PET/CT imaging, Chen et al. (42) concluded that ^99m^Tc-3PRGD_2_ SPECT imaging was useful for the diagnosis and staging of breast cancer, but its sensitivity for detecting small lymph-node metastases appeared to be lower than that of ^18^F-FDG PET/CT imaging (sensitivity: 78.05% vs. 85.37%; p>0.05). Their study also confirmed that the expression of integrin αvβ3 was correlated with ^99m^Tc-3PRGD_2_ uptake (r=0.582; p=0.001) and was especially significantly elevated in patients with HER2-positive and stage III–IV disease (p<0.05).

### Evaluation of Efficacy

Ji et al. (43) explored the application of ^99m^Tc-3PRGD_2_ SPECT imaging to evaluate the efficacy of neoadjuvant chemotherapy in patients with stage II and III breast cancer. Their findings indicated that ^99m^Tc-3PRGD_2_ SPECT imaging could be used to detect changes in tumor pathology and treatment efficacy in this group of patients (sensitivity, specificity, and negative predictive value: 92.9%, 93.3%, and 93.3%, respectively). It was especially advantageous for determining the efficacy of neoadjuvant chemotherapy among patients with HER2-positive breast cancer.

## Esophagus

Esophageal cancer is a major public health concern in China (1). Accurate initial staging of esophageal cancer is the key to selecting the best treatment plan for patients, while complete resection of all cancerous lesions is the key to curing this disease. Lymph-node metastasis is usually the first metastasis to occur in esophageal cancer, and the number of metastatic lymph nodes is significantly negatively correlated with the survival rate (44). In current clinical practice, esophageal cancer is commonly diagnosed using CT and endoscopic ultrasonography, which imposes major constraints on the preoperative assessment of lymph-node metastasis (45, 46). Therefore, it is necessary to identify a more accurate approach for the diagnosis of esophageal cancer and preoperative assessment of lymph-node metastasis.

### Diagnosis and Differential Diagnosis

Gao et al. (47) evaluated the diagnostic value of ^99m^Tc-3PRGD_2_ SPECT imaging for esophageal space-occupying lesions and found that ^99m^Tc-3PRGD_2_ SPECT had clinical potential for the diagnosis of esophageal cancer, with an especially high diagnostic sensitivity for distal lymph-node metastases. Their study also confirmed that ^99m^Tc-3PRGD_2_ uptake in esophageal cancer was unrelated to the pathological type of the tumor (p>0.05) but was significantly positively correlated with the percentage of integrin αvβ3-positive cells (r=0.976). In addition, Chen et al. (48) and Zheng et al. (49) demonstrated that ^99m^Tc-3PRGD_2_ SPECT imaging was useful for diagnosing esophageal space-occupying lesions and assessing their angiogenesis.

### Preoperative Assessment of Lymph-Node Metastasis

By comparing the diagnostic value of ^99m^Tc-3PRGD_2_ SPECT imaging with that of CT for lymph-node metastasis in esophageal cancer, Lv et al. (50) showed that ^99m^Tc-3PRGD_2_ SPECT imaging was more accurate than CT for the preoperative assessment of lymph-node metastasis (sensitivity, specificity, accuracy, positive predictive value, and negative predictive value: 80.95/59.52%, 86.51/73.02%, 85.12/69.64%, 66.67/42.37%, and 93.16/84.40%, respectively) and, therefore, was more helpful for the determination of treatment plans. However, Zheng et al. (49) speculated that ^99m^Tc-3PRGD_2_ SPECT imaging may be less sensitive than ^18^F-FDG PET/CT imaging for the detection of metastatic lesions in small lymph nodes.

## Head and Neck

### Differentiated Thyroid Cancer

Gao et al. (51) assessed the value of ^99m^Tc-3PRGD_2_ SPECT imaging for the monitoring of differentiated thyroid cancer recurrence. Their results indicated that among patients with differentiated thyroid cancer with high serum thyroglobulin levels and negative radioiodine whole-body scan results, ^99m^Tc-3PRGD_2_ SPECT showed higher sensitivity (96.6%) and positive predictive value (93.3%) for monitoring recurrence, while the probability of obtaining a positive SPECT finding was related to the thyroglobulin levels (p=0.006). In addition, a study by Zhao et al. (52) revealed that ^99m^Tc-3PRGD_2_ SPECT imaging could trace radioactive iodine-refractory differentiated thyroid cancer metastases and could be used for the localization and growth evaluation of such lesions. Thus, the study provided a new therapeutic target and new imaging method for monitoring the efficacy of certain anti-angiogenetic therapies.

### Choroidal Melanoma

Yan et al. (53) explored the feasibility of employing ^99m^Tc-3PRGD_2_ SPECT imaging for the diagnosis of choroidal melanoma and monitoring of the tumor response to plaque brachytherapy. Their results indicated that ^99m^Tc-3PRGD_2_ SPECT imaging is a suitable modality for diagnosing choroidal melanoma and evaluating the accuracy of plaque brachytherapy, showing a significantly lower tumor-to-occipital bone ratio at 3 months post-plaque brachytherapy compared to that at baseline. Furthermore, follow up for a minimum of 20 months after plaque brachytherapy suggested that the co-analysis of ^99m^Tc-3PRGD_2_ SPECT imaging with the tumor volume may comprise a promising prognostic predictor for patients with choroidal melanoma.

### Tumors of the Sellar Region

Hou et al. (54) reported one case of combined ^18^F-FDG PET/CT and ^99m^Tc-3PRGD_2_ SPECT/CT imaging for the diagnosis of pituitary metastases where the tumor-to-cerebellum ratio of ^99m^Tc-3PRGD_2_ SPECT/CT was significantly higher than that of ^18^F-FDG PET/CT. Thus, they inferred that ^99m^Tc-3PRGD_2_ SPECT/CT might be useful for differentiating pituitary adenomas from metastases. Further investigations are needed regarding the use of ^99m^Tc-3PRGD_2_ SPECT imaging for the examination of tumors in the sellar region.

### Brain Glioma

Gao et al. (55) conducted a preliminary evaluation of the clinical application of ^99m^Tc-3PRGD_2_ SPECT imaging for brain gliomas and revealed that the tumor T/N ratio (ratio of abnormal nuclide accumulation to little or no nuclide accumulation) was significantly positively correlated with the percentage of integrin αvβ3-positive cells (R^2 =^ 0.9253; p<0.05). Their results, therefore, confirmed the feasibility of diagnosing brain gliomas using ^99m^Tc-3PRGD_2_ SPECT imaging.

## Other

Jin et al. (56) reported a case of benign metastasizing leiomyoma that showed low uptake on ^18^F-FDG PET/CT imaging but significant uptake on ^99m^Tc-3PRGD_2_ SPECT imaging, which they inferred as being attributable to the active angiogenic process in the lesions. Hence, ^99m^Tc-3PRGD_2_ may have potential diagnostic value for benign metastasizing leiomyoma.

## Discussion

Cancer ranks as the leading cause of death and an important barrier to increasing life expectancy worldwide (57). In recent years, PET/CT technology has gradually matured and has been widely used for the diagnosis, staging, and treatment efficacy evaluation of cancer. However, ^18^F-FDG, the most commonly used PET/CT imaging agent, is not specific for malignant tumors, and non-malignant inflammation and infectious disease can also simulate the high intake of malignant lesions (58–61). The tripeptide sequence of RGD can specifically bind to the integrin αvβ3 receptor (10). Accordingly, various radiolabeled RGD-based peptides have been developed for noninvasive imaging of integrin αvβ3 expression (11, 19, 62–69). Compared with the tracers for PET, such as ^18^F-Ga-lacto-RGD (11, 62), ^18^F-FPPRGD_2_ (63, 64), and ^68^Ga-RGD_2_ (19, 67), ^99m^Tc-3PRGD_2_ is superior as an easy-labeling, cost-effective SPECT tracer using the generator-produced ^99m^Tc and broadly available SPECT system, rather than relying on an onsite cyclotron and expensive PET system (26). Moreover, the preparation procedure for ^99m^Tc-3PRGD_2_ is simple, efficient, and reproducible, allowing a kit formulation and easy availability for routine clinical use (15, 70), which can better meet clinical needs.

In the past decade, ^99m^Tc-3PRGD_2_ SPECT for integrin αvβ3 receptor imaging has made rapid progress in clinical research, and its application for various tumor lesions has been widely demonstrated. ^99m^Tc-3PRGD_2_ SPECT imaging can be applied to the diagnosis of tumors positive for the integrin αvβ3 receptor, determination of the location and extent of tumors and metastases, monitoring of postoperative residual or recurrent lesions, auxiliary diagnosis of neovascular density, and efficacy and prognostic evaluation of tumor anti-neovascular therapy. With the progress and success of ^99m^Tc-3PRGD_2_ SPECT imaging for a wide range of clinical applications, it provides a better understanding of the pathophysiological changes and therapeutic responses of lesions, and it can be expected to have a bright prospect for its future use. Considering the good diagnostic performance of ^99m^Tc 3PRGD2 SPECT imaging, it may be possible to non-invasively evaluate some tumoral histopathological subtypes, such as those of non-small cell lung cancer. Concurrently, immunotherapy for malignant tumors is one of the most popular topics at present. It is difficult to predict the response after immunotherapy and evaluate the efficacy of immunotherapy with traditional imaging, but molecular imaging based on the integrin αvβ3 receptor may be an effective means for predicting and evaluating immunotherapy. In addition, non-oncology applications of RGD peptide-based SPECT imaging have been reported in the literature, showing exciting prospects and may become a hot topic for future research (71).

Unfortunately, although rapid progress has been recently made in tumor-related applications of ^99m^Tc-3PRGD_2_ SPECT imaging, most advances have remained at the clinical trial stage and have numerous shortcomings. The main limitations are the low sensitivity and spatial resolution of the SPECT/CT scanner and the substantial impact of attenuation and scattering, resulting in lower image quality. However, these deficiencies are expected to be resolved to a great extent with the introduction of next-generation SPECT/CT scanners.

In this review, we presented an overview of the current clinical development of ^99m^Tc-3PRGD_2_ SPECT imaging in oncology and further discussed its potential future clinical applications. With further research and clinical transformation, as well as update and improvement of SPECT imaging equipment and scanning sequences, ^99m^Tc-3PRGD_2_ SPECT imaging undoubtedly has marked application prospects, and we await further breakthroughs on several grounds, including early diagnosis of tumors, dynamic evaluation of tumor treatment efficacy and prognosis, and individualized guidance for molecular targeted therapy.

## Author Contributions

LX drafted the manuscript. JX contributed with discussions and critical revision of the manuscript. Both authors reviewed, commented, and revised the manuscript. Both authors contributed to the article and approved the submitted version.

## Funding

This work was supported by the 345 Talent Project from Shengjing Hospital of China Medical University [grant/award number M0441].

## Conflict of Interest

The authors declare that the research was conducted in the absence of any commercial or financial relationships that could be construed as a potential conflict of interest.

## Publisher’s Note

All claims expressed in this article are solely those of the authors and do not necessarily represent those of their affiliated organizations, or those of the publisher, the editors and the reviewers. Any product that may be evaluated in this article, or claim that may be made by its manufacturer, is not guaranteed or endorsed by the publisher.
